# Inhibition of TRF2 Leads to Ferroptosis, Autophagic Death, and Apoptosis by Causing Telomere Dysfunction

**DOI:** 10.1155/2023/6897268

**Published:** 2023-04-18

**Authors:** Qiuhui Yang, Ziyang Nie, Yukun Zhu, Mingying Hao, Siqi Liu, Xuelu Ding, Feng Wang, Fei Wang, Xin Geng

**Affiliations:** ^1^Department of Biochemistry and Molecular Biology, School of Basic Medical Sciences, Tianjin Medical University, Tianjin 300070, China; ^2^Key Laboratory of Immune Microenvironment and Disease (Ministry of Education), Tianjin Medical University, Tianjin 300070, China; ^3^School of Life Sciences, Central China Normal University, Hubei Province, China; ^4^Fuyang Hospital Affiliated to Anhui Medical University, Anhui Province 236000, China; ^5^Department of Neurology, Tianjin Neurological Institute, Tianjin Medical University, General Hospital, Tianjin 300052, China; ^6^Department of Genetics, School of Basic Medical Sciences, Tianjin Medical University, Tianjin 300070, China; ^7^Department of Neurology, General Hospital, Tianjin Medical University, Tianjin 300052, China

## Abstract

**Background:**

Gastric cancer (GC) is an aggressive malignancy with a high mortality rate and poor prognosis. Telomeric repeat-binding factor 2 (TRF2) is a critical telomere protection protein. Emerging evidence indicates that TRF2 may be an essential treatment option for GC; however, the exact mechanism remains largely unknown.

**Objective:**

We aimed to explore the role of TRF2 in GC cells. The function and molecular mechanisms of TRF2 in the pathogenesis of GC were mainly discussed in this study.

**Methods:**

Relevant data from GEPIA and TCGA databases regarding TRF2 gene expression and its prognostic significance in GC samples were analyzed. Analysis of 53BP1 foci at telomeres by immunofluorescence, metaphase spreads, and telomere-specific FISH analysis was carried out to explore telomere damage and dysfunction after TRF2 depletion. CCK8 cell proliferation, trypan blue staining, and colony formation assay were performed to evaluate cell survival. Apoptosis and cell migration were determined with flow cytometry and scratch-wound healing assay, respectively. qRT-PCR and Western blotting were carried out to analyze the mRNA and protein expression levels after TRF2 depletion on apoptosis, autophagic death, and ferroptosis.

**Results:**

By searching with GEPIA and TCGA databases, the results showed that the expression levels of TRF2 were obviously elevated in the samples of GC patients, which was associated with adverse prognosis. Knockdown of TRF2 suppressed the cell growth, proliferation, and migration in GC cells, causing significant telomere dysfunction. Apoptosis, autophagic death, and ferroptosis were also triggered in this process. The pretreatment of chloroquine (autophagy inhibitor) and ferrostatin-1 (ferroptosis inhibitor) improved the survival phenotypes of GC cells.

**Conclusion:**

Our data suggest that TRF2 depletion can inhibit cell growth, proliferation, and migration through the combined action of ferroptosis, autophagic death, and apoptosis in GC cells. The results indicate that TRF2 might be used as a potential target to develop therapeutic strategies for treating GC.

## 1. Introduction

Gastric cancer remains a significant clinical problem worldwide. With over 1 million estimated new cases in 2018, gastric cancer is the fifth most common cancer. Gastric cancer is estimated about 783,000 people deaths globally in 2018, making it the third leading cause of cancer-related death [1]. It is a molecularly and phenotypically highly heterogeneous disease [2]. Gastric cancer represents a major burden of society, and treatment of this disease needs to be improved [3]. For early-stage gastric cancer, there is a greater emphasis placed on the tumour resection. A comprehensive treatment approach including traditional radiotherapy and chemotherapy, targeted therapy, and immunotherapy was adopted for intermediate-stage gastric cancer. Targeted therapy combined with chemotherapy has become the first-line treatment guideline for advanced gastric cancer. The choice of targeted drugs for gastric cancer is relatively limited. Trastuzumab, a recombinant humanized IgG1 monoclonal antibody directed against the extracellular domain of HER2, is the only first-line targeted therapy drug. However, only 20% or less of gastric cancer patients are HER-2 positive [4, 5]. Although significant efforts have been made to treat gastric cancer effectively, the incidence and mortality of gastric cancer remain high. Individualized treatment strategies and deeper molecular mechanisms need to be explored [6, 7].

Telomeres, regions of repetitive DNA sequences at the ends of chromosomes, are composed of tandem DNA repeats and shelterin complexes [8]. Shelterin specifically binds to telomeric DNA and is composed of six proteins: TRF1, TRF2, RAP1, POT1, TIN2, and TPP1 [9]. The shelterin complex protects the ends of chromosomes from the DNA damage response (DDR) and maintains genome stability [10]. Maintaining the normal structure and function of telomeres plays a vital role in the stability of the genome. Telomere dysfunction can lead to genome disorders, which in turn lead to cell apoptosis. Researchers have paid increasing attention to the role of telomeres in tumours [11, 12]. The primary function of TRF2, one of the core proteins of shelterin, is to prevent telomeres from being recognized as double-strand breaks (DSBs) by promoting the formation of telomere terminal T loops, thereby maintaining the stable function of telomeres [13, 14]. It has been reported that inhibition of TRF2 leads to chromosome end fusion, growth arrest, and apoptosis [15, 16]. Apoptosis is a kind of programmed cell death that plays a key role in the physiology and pathophysiology of multicellular organisms [17]. TRF2 not only has telomere protection-related functions but also acts as a DNA damage repair factor to promote the survival of cancer cells. Hence, TRF2 has been considered a potential oncogene [18, 19]. A comprehensive and mechanistic understanding of the cellular impact of TRF2 is necessary for further exploration.

Ferroptosis is an iron-dependent form of regulated cell death characterized by mitochondrial shrinkage, iron accumulation, and excess lipid peroxidation [20]. Several studies have implicated the contribution of ferroptosis in the progression of multiple diseases [21], including gastric cancer [22]. Cystine uptake by the cystine/glutamate transporter system xc- represents the upstream event of ferroptosis under extracellular oxidative conditions [23]. p53 can enhance ferroptosis by inhibiting the expression of the cystine/glutamate antiporter SLC7A11 (also commonly known as xCT) or by enhancing the expression of SAT1 and GLS2 [24]. Tanshinone IIA was also found to upregulate p53 expression and downregulate xCT expression, thereby suppressing the proliferation of gastric cancer via inducing p53 upregulation-mediated ferroptosis [25]. TRF2 deletion provokes the induction of an acute DDR at telomeres, leading to the activation of p53 signalling pathways and programmed cell death [19]. However, whether TRF2 depletion causes ferroptosis in gastric cancer cells remains unclear.

Autophagy is a catabolic process in which cytoplasmic components are delivered to vacuoles or lysosomes for degradation and nutrient cycling [26]. This degradation process is critical to maintaining cellular homeostasis, as it rids the cell of either excess or damaged organelles, aggregated proteins, or pathogens [27]. However, excessive autophagy can inhibit the growth and proliferation of cells. The loss of autophagy function is necessary for cancer cell proliferation [28, 29]. Telomere dysfunction can cause cell autophagy through the cyclic GMP-AMP synthase-stimulator of interferon genes (cGAS-STING) signalling pathway. The G-quadruplex ligand SYUIQ-5 triggered potent telomere damage through TRF2 delocalization from telomeres and eventually induced autophagic cell death in cancer cells [30]. In our present study, we explored whether inhibiting the expression level of TRF2 in gastric cancer could limit the growth, proliferation, and migration of gastric cancer cells by inducing autophagic death.

Overall, we aimed to explore whether TRF2 depletion in gastric cancer cells could inhibit cell growth, proliferation, and migration through the combined action of ferroptosis, autophagic death, and apoptosis and whether inhibition of ferroptosis or autophagy would augment the recovery of cell growth, proliferation, and migration. These data will provide new targets and avenues for treating of gastric cancer.

## 2. Materials and Methods

### 2.1. Bioinformatics Analysis

The mRNA expression levels of TRF2 in normal tissues and gastric cancer tissues were searched in the GEPIA (http://gepia.cancer-pku.cn/) and TCGA (http://portal.gdc.cancer.gov) databases, respectively. The relationship between the expression level of TRF2 mRNA and the survival time of patients from which gastric cancer samples and normal samples were collected was determined using the cBioPortal database (http://www.cbioportal.org/), and the statistical significance was analyzed by GraphPad Prism 5 software. The STRING database (http://string-db.org/) was used to query the network of proteins interacting with TRF2, p53, SLC7A11, and glutathione peroxidase 4 (GPX4). The STRING database can be used to annotate the structure, function, and evolutionary properties of proteins. It can also explore and predict interacting protein networks and provide new research directions for future experiments.

### 2.2. Cell Culture and Plasmid Amplification

The human gastric cancer cell line BGC-823 was from ATCC. Cells were cultured in Dulbecco's modified Eagle's medium (DMEM; 06-1055-57-1ACS Biological Industries) supplemented with 10% foetal bovine serum (FBS; 04-001-1ACS, Biological Industries) in a humidified chamber with 5% CO_2_ at 37°C. To establish a stable TRF2 knockdown cell line, lentiviruses carrying TRF2-targeting shRNA (GenePharma) were transduced into BGC-823 cells along with 5 *μ*g/mL polybrene (H8761, Solarbio), and cells were selected for with 1 *μ*g/mL puromycin (A1113803, Thermo Fisher). Plasmid transfection was carried out using PEI (919012-100MG, Sigma-Aldrich), as recommended by the manufacturer. TRF2 shRNA sequences were as follows: shRNATRF2-1 sense, 5′-CCGGGCGCATGACAATAAGCAGATTCTCGAGAATCTGCTTATTGTCATGCGCTTTTT-3′, antisense, 5′-AATTAAAAAGCGCATGACAATAAGCAGATTCTCGAG AATCTGCTTATTGTCATGCGC-3′; and shRNATRF2-2 sense, 5′-CCGGCATTGGAATGATGACTCTGAACTCGAGTTCAGAGTCATCATTCCAATGTTTTT-3′, antisense 5′-AA TTAAAAACATTGGAATGATGACTCTGAACTCGAGTTCAGAGTCATCATTCCAATG-3′. The TRF2 gene was amplified by PCR from the cDNA of BGC-823 cells. The PCR products were purified with a gel extraction kit (DP209-03, TIANGEN). Then, the restriction endonucleases EcoRI and AgeI were used to digest the purified products and the pLKO.1-TRC cloning vector. After purification, the products and vector were ligated using T4 DNA ligase (e0879, Takara). Finally, the plasmids were amplified in DH5*α E. coli* cells and identified by sequencing.

### 2.3. Quantitative Real-Time PCR (qRT-PCR)

Total RNA was isolated using a total RNA extraction kit (DP419, TIANGEN) according to the manufacturer's instructions. One microgram of RNA was used as a template to synthesize cDNA using the GoScript Reverse Transcription System (PRA5000, Promega). All qPCRs were carried out using the Roche LightCycler System. The primers used in this study are from GenBank (https://pga.mgh.harvard.edu/primerbank/index.html) and are as follows: GAPDH, 5′-GTCTCCTCTGACTTCAACAGCG-3′ and 5′-ACCACCCTGTTGCTGTAGCCAA-3′; TRF2, 5′-GTGGAAAAGCCACCCAGAGAAC-3′ and 5′-TGCAAAGGCTGCCTCAGAATCC-3′; GPX4, 5′-ACAAGAACGGCTGCGTGGTGAA-3′ and 5′-GCCACACACTTGTGGAGCTAGA-3′; SLC7A11, 5′-TCCTGCTTTGGCTCCATGAACG-3′ and 5′-AGAGGAGTGTGCTTGCGGACAT-3′; p62, 5′-ACGCAGAACAGAGTTACGAAGGC-3′ and 5′-CCAGTCATCTTGTCCGTAGGCTTC-3′; and ATG5, 5′-GCAAGCCAAGGAGGAGAAGATTCC-3′ and 5′-GTGTCTCAGCGAAGCAGTGGTG-3′. Each sample was assayed in triplicate.

Relative mRNA levels were determined using the 2^−*ΔΔ*Ct^ method.

### 2.4. Western Blotting

Cells were lysed in RIPA lysis buffer (R0020, Solarbio) supplemented with phosphatase inhibitor cocktail (4906837001, Roche) and protease inhibitor cocktail (11836170001, Roche). Protein contents were measured with the BCA Protein Assay (23227, Thermo Fisher Scientific). Equal amounts of protein extracts were separated on an SDS–PAGE gel, followed by electrotransfer onto a PVDF membrane (EZWB05-ISEQ00010-1, Millipore). The blots were subsequently incubated for 2 h in a blocking buffer. The membranes were incubated at 4°C overnight with monoclonal antibodies, followed by corresponding secondary anti-rabbit or anti-mouse antibodies for 2 h. The antibodies used for Western blotting were as follows: SLC7A11 (A2413, ABclonal), GPX4 (A1933, ABclonal), ATG5 (10181-2-AP, Proteintech), LC3 (14600-1-AP, Proteintech), *β*-actin (81115-1-RR, Proteintech), GAPDH (10494-1-AP, Proteintech), TRF2 (22020-1-AP, Proteintech), secondary anti-rabbit antibody (SA00001-2, Proteintech), and anti-mouse antibodies (SA00001-1, Proteintech). The proteins were detected with ECL Blotting Detection Reagents (32106, Thermo Fisher Scientific).

### 2.5. Trypan Blue Staining

Cell activity was detected with trypan blue staining. In brief, the medium in six-well plates was discarded. Trypsin was added to digest the cells for 90 sec, and then, the trypsin was discarded. A total of 900 *μ*L of complete medium and 100 *μ*L of 0.4% trypan blue dye were added to the six-well plates; they were shaken well and then incubated for 3 minutes. Finally, 10 *μ*L of the trypan blue cell suspension (C0040, Solarbio) was dropped onto a special cell counting plate, and the cells were counted under an ordinary light microscope (MARIENFELD). Cell viability was expressed as a percentage of the DMSO-treated control.

### 2.6. Cell Proliferation Assay with Cell Counting Kit-8 (CCK-8)

A CCK-8 assay was used to evaluate cell proliferation. Cells were counted and seeded into 96-well plates. The Cell Counting Kit-8 (CCK-8; C3007, Beyotime Institute of Biotechnology) was used to determine the cell proliferation rate according to the manufacturer's instructions. Briefly, after 0, 24, 48, and 72 h of growth, the cells were incubated in CCK-8 reagent at 37°C for 2 h. Then, absorbance at 450 nm was measured using a microplate reader. Each test was repeated in triplicate under the same conditions.

### 2.7. Scratch-Wound Healing Assay

Three parallel lines were drawn across the bottom of the wells in a six-well plate, and the cells were spread onto the plates. The cells were scratched in the direction perpendicular to the drawn parallel lines using a 200 *μ*L pipette tip. The old medium was discarded, and 1 mL PBS was added for washing. A total of 2 mL of serum-free medium was added, and then, the cells were observed and photographed with a fluorescence microscope. The scratch width was measured 48 h after the first scratch was made, and the healing percentage and scratch width recovery percentage between cells in each well were compared. The wound surface area was quantified by ImageJ software.

### 2.8. Colony Formation Analysis

BGC-823 cells were slowly washed twice with deionized water, then 4% tissue cell fixative (BL539A, Biosharp) was added, and the cell culture dish was allowed to stand for 20 min. The cells were washed with deionized water three times, a 0.5% crystal violet dye solution (E607309-0100, Sangon Biotech) was added to completely infiltrate the cells, and the cells were allowed to stand at room temperature for 30 min. Deionized water was added, and the cell culture dish was slowly washed three times on a shaker for 2 min each time. Finally, the cell culture dish was placed upside down on filter paper, the water was drained, and the results were analyzed with ImageJ. Each experiment was repeated in triplicate under the same conditions.

### 2.9. Analysis of 53BP1 Foci at Telomeres by Immunofluorescence

BGC-823 cells were fixed with paraformaldehyde (E672002-0500, Sangon), washed, and permeabilized in 0.5% Triton X-100 (T8787-100ML, Sigma). After blocking, the cells were immunostained with 53BP1 antibody (1 : 1000; ab175933, Abcam) and then immunostained with Alexa Fluor 555-labeled anti-rabbit IgG fluorescent antibody (1 : 2000, ab150078, Abcam). Prepared PBST solution was added for washing, followed by dehydration with ethanol. Finally, 4′,6-diamidino-2-phenylindole (DAPI; H-1200-10, Vectorlabs) was added and sealed, and the results were observed under a fluorescence microscope.

### 2.10. Metaphase Spreads and Telomere Fluorescence In Situ Hybridization (FISH) Analysis

Telomere dysfunction-induced foci (TIFs) were assessed by metaphase spreads and telomere-specific FISH to detect telomere dysfunction. Cy3-labeled telomere-specific peptide nucleic acid (PNA) was applied to the sample, denatured by incubation for 4 min at 83°C, and hybridized in the dark at room temperature for 2 hours. Slides were then rinsed in PBST, followed by the application of fluorescent secondary antibody conjugated to Alexa Fluor 488 (diluted 1 : 100; A11034, Life Technologies, Grand Island, NY) and incubation at room temperature for 30 min. Slides were then counterstained with DAPI, and the results were observed under a fluorescence microscope.

### 2.11. Cell Apoptosis Assay

An Annexin V-FITC and PI apoptosis detection kit (556547, BD Biosciences) was used according to the manufacturer's directions. Briefly, cells were resuspended at 1 × 10^5^ cells/mL in 100 *μ*L of binding buffer. Annexin V-FITC (5 *μ*L) and PI (5 *μ*L and 20 *μ*g/mL) were added to the cell suspension, followed by incubation at room temperature for 10  minutes in the dark and then mixing with 1 mL of binding buffer. A total of 1 × 10^6^ cells/sample was analyzed on a FACSCalibur.

### 2.12. Statistical Analysis

Each test in this study was repeated in triplicate under the same conditions. Quantitative data are expressed as means ± SD of three independent experiments. Statistical significance was determined using one-way ANOVA and two-tailed Student's *t*-test. Data analysis was carried out using GraphPad Prism 7.0 (GraphPad Software). *P* values of < 0.05 were considered statistically significant.

## 3. Results

### 3.1. TRF2 Is an Oncogene in Gastric Cancer, and TRF2 Depletion Inhibits the Growth, Proliferation, and Migration of Gastric Cancer Cells

**Figure 1:**
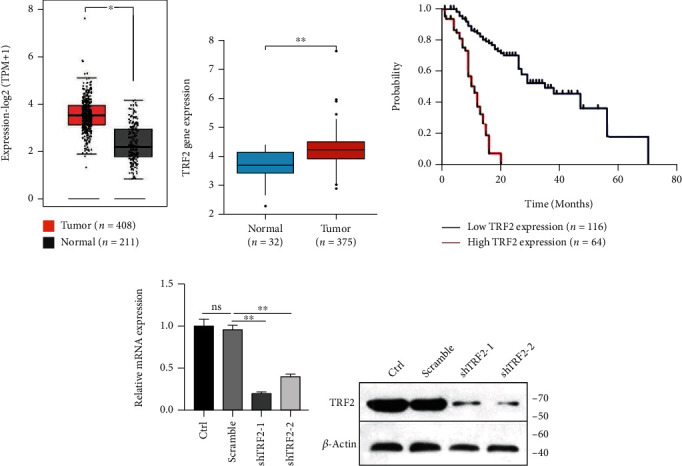
Analysis of TRF2 mRNA expression levels in gastric cancer using bioinformatic databases and TRF2 shRNA knockdown. (a, b) Box plots of the TRF2 mRNA expression levels in gastric cancer tissues and corresponding normal tissues obtained by searching the GEPIA database (a) and TCGA database (b). (c) Survival analysis of gastric cancer patients with high levels of TRF2 mRNA expression and with low levels of TRF2 mRNA expression. (d, e) TRF2 mRNA expression levels and protein expression levels in TRF2 shRNA-transfected cells and scramble shRNA control cells. Each test was repeated in triplicate under the same conditions, and representative data are shown. Comparisons were performed using Student's *t*-test and one-way ANOVA. ns: not significant. ^∗^*P* < 0.05 and ^∗∗^*P* < 0.01.

We used a bioinformatic method to predict whether TRF2 could be used as an oncogene in gastric cancer. First, we compared the expression level of TRF2 mRNA between gastric cancer tissues and corresponding normal gastric mucosa tissues by searching the GEPIA and TCGA databases. As shown in Figures 1(a) and 1(b), TRF2 mRNA in gastric cancer tissues was significantly higher than in normal tissues. This finding indicates that TRF2 is overexpressed in gastric cancer. To predict whether TRF2 affects the survival and prognosis of patients with gastric cancer, we used the clinical information obtained from the cBioPortal database for survival analysis. As shown in Figure 1(c), the survival time of gastric cancer patients with low TRF2 mRNA expression was significantly longer than that of patients with high TRF2 expression.

**Figure 2:**
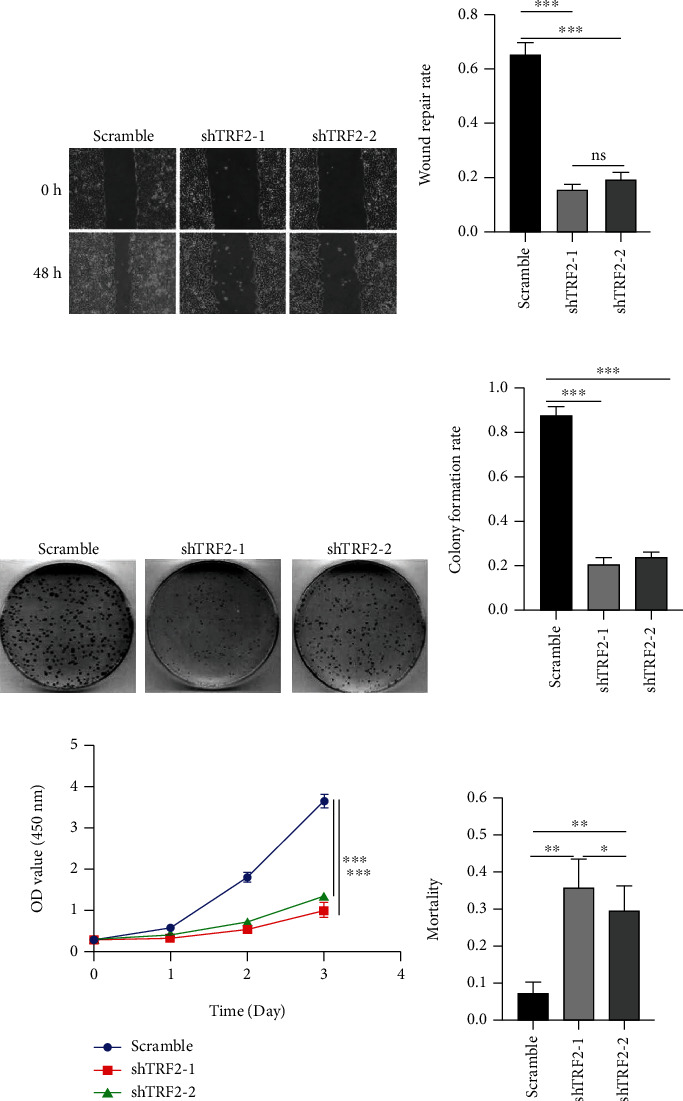
Effects of TRF2 knockdown on the growth, proliferation, and migration of gastric cancer cells. (a, b) The cell migration ability was detected by scratch-wound healing assays. (c, d) Cell proliferation was detected by colony formation assays. (e) CCK-8 assay growth curves of TRF2 knockdown cells and control cells. (f) Cell viability determined by the trypan blue staining assay. Each test was repeated in triplicate under the same conditions, and representative data are shown. Comparisons were performed using Student's *t*-test and one-way ANOVA. ns: not significant. ^∗^*P* < 0.05, ^∗∗^*P* < 0.01, and ^∗∗∗^*P* < 0.001.

Because we have demonstrated that TRF2 may be an important oncogene of gastric cancer using bioinformatic databases, we next explored the effects of TRF2 on the growth, proliferation, and migration of gastric cancer cells. We designed two plasmids containing different sequences of TRF2-shRNA and infected BGC-823 cells with these lentiviral vectors. To verify the efficiency of TRF2 knockdown by two plasmids, we conducted qRT-PCR and Western blot experiments. As shown in Figures 1(d) and 1(e), the expression levels of TRF2 mRNA and protein in the experimental group were significantly lower than those in the control group, which indicated that both plasmids could be used in subsequent molecular biology experiments. We compared the proliferation, survival, and migration of gastric cancer cells between the control group and the stable knockdown TRF2 group by CCK-8, colony formation, trypan blue staining, and scratch-wound healing assays. As shown in Figures 2(a) and 2(b), knockdown of TRF2 significantly inhibited the migration level of gastric cancer cells. The results in Figures 2(c)–2(e) show that knockdown of TRF2 could evidently restrict the growth and proliferation of gastric cancer cells. The results of the trypan blue staining assay (Figure 2(f)) showed that reducing the expression level of TRF2 in gastric cancers could obviously inhibit the viability of the cells. The above results suggest that specific knockdown of TRF2 has a remarkable inhibitory effect on the growth, proliferation, and migration of gastric cancer cells, and TRF2 may play an important role in the occurrence and development of gastric cancer.

### 3.2. TRF2 Knockdown in Gastric Cancer Cells Causes Telomere Dysfunction

**Figure 3:**
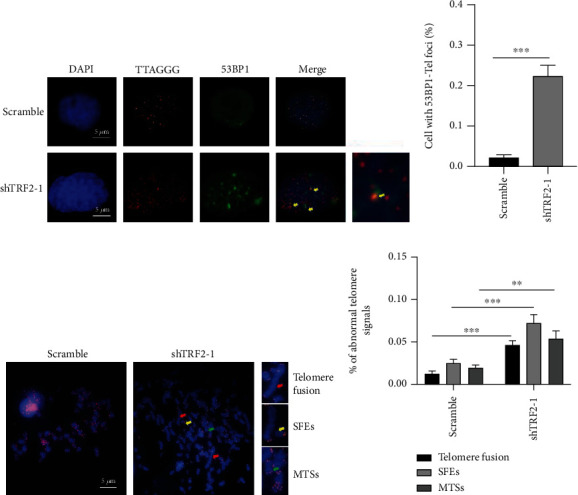
Effect of TRF2 knockdown on telomere dysfunction in gastric cancer cells. (a, b) Analysis of 53BP1 foci at telomeres by immunofluorescence. The red dots represent telomere signals, and the green dots represent 53BP1 signals. The yellow arrows represent 53BP1 foci at the telomeres. Scale bars, 5 *μ*m. (c, d) The results of metaphase spreads and telomere fluorescence in situ hybridization (FISH) analyses. At least 10 mitotic phases were counted in each group, and the total number of chromosomes was more than 300. SFEs: telomere signal free ends; MTSs: multiple telomere signals. Scale bars, 5 *μ*m. Comparisons were performed using Student's *t*-test and one-way ANOVA. ns: not significant. ^∗∗^*P* < 0.01 and ^∗∗∗^*P* < 0.001.

TRF2 is one of the key telomere protection proteins. Telomere dysfunction caused by TRF2 inhibition in various types of cancer cells will activate the DDR, which will lead to abnormal telomere end signals [10, 11]. To determine whether knockdown of TRF2 leads to the same outcome in gastric cancer cells, we detected telomere dysfunction-induced foci (TIFs) in gastric cancer cells by immunofluorescence of 53BP1 foci at telomeres and telomere FISH assays. The results showed that knockdown of TRF2 significantly upregulated the colocalization of the DNA damage marker 53BP1 with telomeres (Figures 3(a) and 3(b)). The results of telomere-specific FISH (Figures 3(c) and 3(d)) showed that knockdown of TRF2 in gastric cancer cells could induce obvious abnormal signals at telomere ends, including telomere signal free ends (SFEs) and multiple telomere signals (MTSs). Furthermore, the frequency of chromosome end-to-end fusions significantly increased was observed in depletion of TRF2. These results suggest that specific inhibition of TRF2 in gastric cancer cells can cause significant telomere dysfunction and chromosome end-to-end fusions.

### 3.3. Knockdown of TRF2 Promotes Autophagic Death and Apoptosis

**Figure 4:**
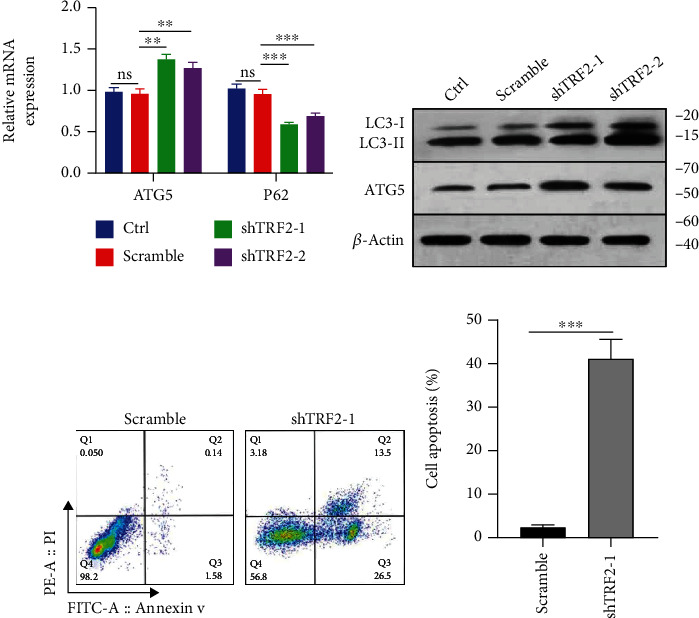
Effects of TRF2 knockdown on autophagy and apoptosis in gastric cancer cells. (a, b) The mRNA and protein expression levels of autophagy markers were measured by qRT-PCR and Western blotting, respectively. (c, d) The level of apoptosis was detected by flow cytometry. Each test was repeated in triplicate under the same conditions, and representative data are shown. Comparisons were performed using Student's *t*-test and one-way ANOVA. ns: not significant. ^∗∗^*P* < 0.01 and ^∗∗∗^*P* < 0.001.

It has been reported that telomere dysfunction can lead to autophagic death, apoptosis, and other forms of death. To further verify whether the telomere dysfunction caused by TRF2 knockdown in gastric cancer cells can induce autophagic death and apoptosis, we compared the levels of autophagy and apoptosis between control and TRF2 knockdown gastric cancer cells by qRT-PCR, Western blotting, and flow cytometry. The mRNA level of autophagy-related 5 (ATG5) was significantly increased, and p62 was significantly decreased (Figure 4(a)) in the TRF2 knockdown cells. ATG5 is a molecule involved in the activated autophagy machinery, while decreased levels of p62, an autophagic flux marker, can be observed when autophagy is induced. The protein levels of LC3-II (phosphatidylethanolamine-conjugated form of MAP1LC3B) and ATG5 were significantly increased (Figure 4(b)) in the TRF2 knockdown cells. The quantitative analysis results showed that the expression of LC3 and ATG5 protein increased by more than 50% compared with the scramble group. Figures 4(c) and 4(d) show that TRF2 knockdown can also induce apoptosis. Therefore, these results show that telomere dysfunction caused by TRF2 inhibition can promote autophagic death and apoptosis in gastric cancer cells.

### 3.4. Inhibition of TRF2 Simultaneously Boosts Ferroptosis

**Figure 5:**
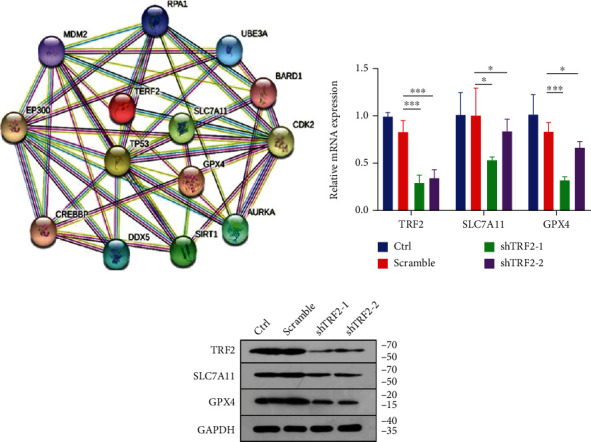
Effects of TRF2 knockdown on ferroptosis in gastric cancer cells. (a) The TRF2, p53, and ferroptosis-related protein interaction network was determined using the STRING database. (b, c) The mRNA and protein expression levels of ferroptosis markers were measured by qRT-PCR and Western blotting, respectively. Each test was repeated in triplicate under the same conditions, and representative data are shown. Comparisons were performed using Student's *t*-test and one-way ANOVA. ns: not significant. ^∗^*P* < 0.05 and ^∗∗∗^*P* < 0.001.

Ferroptosis is a new type of programmed cell death that is iron-dependent and different from apoptosis and autophagic death. The main sign of ferroptosis is the decreased expression of SLC7A11 and GPX4. A large number of studies have shown that p53 inhibits the expression of SLC7A11 and promotes ferroptosis. It is well known that telomere DNA damage can activate p53 pathway. Therefore, to understand the relationship between TRF2, p53, and ferroptosis-related proteins (SLC7A11 and GPX4), the STRING database was used to analyze their protein interaction network. Some important proteins may play roles, including AURKA, BARD1,CDK2, CREBBP, DDX5, EP300, MDM2, RPA1, SIRT1, and UBE3A, which were found to be a part of the TRF2, p53, and ferroptosis-related protein network (Figure 5(a) and Table 1). To further clarify the effect of TRF2 knockdown on ferroptosis in gastric cancer cells, we measured the expression levels of ferroptosis-related markers in TRF2 knockdown gastric cancer cells at the mRNA and protein levels. As shown in Figures 5(b) and 5(c), knockdown of TRF2 significantly decreased the expression levels of SLC7A11 and GPX4 in gastric cancer cells. The quantitative analysis results showed that the expression of SLC7A11 and GPX4 protein decreased by more than 50% compared with the scramble group. These results suggest that depletion of TRF2 can facilitate ferroptosis in gastric cancer cells.

### 3.5. Autophagic Death and Ferroptosis Inhibitors Restore the Inhibition of Cell Growth, Proliferation, and Migration Caused by TRF2 Depletion

**Figure 6:**
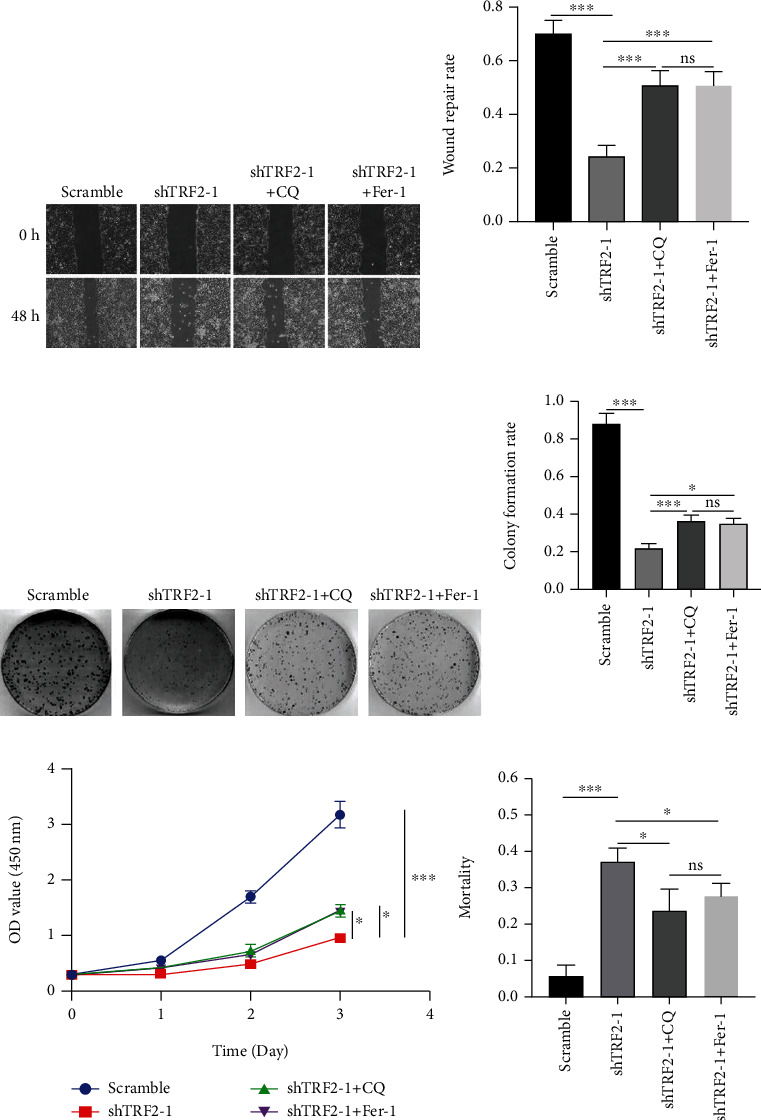
Effects of pretreatment with autophagy and ferroptosis inhibitors on the growth, proliferation, and migration of TRF2 knockdown gastric cancer cells. (a, b) The cell migration ability was detected by scratch-wound healing assays. (c, d) Cell proliferation was detected by colony formation assays. (e) CCK-8 assay growth curves of TRF2 knockdown cells and control cells. (f) Cell viability determined by the trypan blue staining assay. Each test was repeated in triplicate under the same conditions, and representative data are shown. CQ: chloroquine; Fer-1: ferrostatin-1. Comparisons were performed using Student's *t*-test and one-way ANOVA. ns: not significant; ^∗^*P* < 0.05 and ^∗∗∗^*P* < 0.001.

To further prove that the decline in the growth, proliferation, and migration ability of gastric cancer cells caused by knockdown of TRF2 is related to autophagy and ferroptosis, we used chloroquine (CQ), an autophagy inhibitor, and ferrostatin-1 (Fer-1), a ferroptosis inhibitor, to pretreat TRF2 knockdown gastric cancer cells. The growth, proliferation, and migration of gastric cancer cells were detected by CCK-8, colony formation, and trypan blue staining assays. The results are shown in Figures 6(a)–6(f). Pretreatment with the autophagy inhibitor CQ and the ferroptosis inhibitor Fer-1 obviously reversed the inhibited growth, proliferation, and migration ability of gastric cancer cells induced by the knockdown of TRF2. Therefore, telomere dysfunction caused by knockdown of TRF2 can indeed inhibit gastric cancer cell growth and survival through the autophagic death and ferroptosis pathways.

## 4. Discussion

Gastric cancer is the most common malignant tumour of the digestive system, and gastric cancer mortality ranks among the top of all tumour types in China [31, 32]. To date, the annual number of patients diagnosed with and dying of gastric cancer is still increasing, especially in patients with *Helicobacter pylori* infection [33]. Thus, there is an urgent need to develop effective therapies for gastric cancer. The role of telomeres in tumours has attracted increasing attention from researchers. The telomere protective protein shelterin maintains telomere stability and plays an important role in the occurrence and development of tumours. TRF2, one of the core components of shelterin, can protect telomeres and prevent telomere terminal fusion and the DDR. Inhibition of TRF2 can lead to apoptosis mediated by p53 and ATM [34]. The small molecules, curcusone C and quindoline derivative CK1-14, could induce telomeric DNA-damage response in cancer cells through inhibition of TRF2. These small molecules could inhibit tumour cell proliferation and cause cell cycle arrest, resulting in cell apoptosis [35, 36]. Lentiviral vectors and RNA interference technology determine the impact of targeted depletion of TRF2 on the proliferation and tumour-generating activity of human glioblastoma stem cells (GSCs). Targeting TRF2 significantly increased the survival of mouse bearing GSC xenografts [37]. In this regard, we explored the expression levels of TRF2 mRNA in gastric cancer by bioinformatic methods. Our results showed that the mRNA expression levels of TRF2 in gastric cancer samples were higher than those in healthy controls. The survival time of patients with high TRF2 expression was shorter than that of patients with low TRF2 expression. We found that knockdown of TRF2 could inhibit gastric cancer cell growth, proliferation, and migration. These data suggest that TRF2 might promote the progression of gastric cancer.

Apoptosis is a type of programmed cell death that plays key roles in the physiology and pathophysiology of multiple cellular organisms [17]. Knockdown of TRF2 in healthy T cells increased in telomeric DNA damage and T cell apoptosis. In contrast, overexpression of TRF2 in HCV T cells alleviated telomeric DNA damage and T cell apoptosis [38]. Our present research found that knockdown of TRF2 significantly exacerbated apoptosis in gastric cancer cells.

Autophagy is usually described as a double-edged sword in different states of cancer. Cytoprotective autophagy can provide cancer cells with the ability to resist injury. Conversely, cytotoxic autophagy is considered an independent mechanism of cell death [39], and that excessive stimulation of autophagy through overexpression of beclin1 suppresses tumorigenesis [40]. The activation of autophagy is critical for cell death, as its suppression promotes bypass of crisis, continued proliferation, and accumulation of genome instability. Prolonged stress and progressive autophagy can also eventually lead to cell death [41]. Telomere dysfunction specifically triggers autophagy, indicating that the telomere-driven autophagy pathway is not induced by intrachromosomal breaks. Telomeric DNA damage generates cytosolic DNA species with fragile nuclear envelopes. The cytosolic chromatin fragments activate the cGAS-STING pathway and engage the autophagy machinery [42]. Telomere deprotection by TRF2 depletion was sufficient to activate autophagy independently of replicative crisis [43]. Our present research found that inhibition of TRF2 in gastric cancer cells could significantly increase the expression of autophagy markers (ATG5 and LC3-II) and decrease the expression of the autophagic flux marker p62, indicating that TRF2 deficiency facilitated the process of autophagy in gastric cancer cells. Additionally, pretreatment with chloroquine significantly reversed the inhibition of cell growth, proliferation, and migration caused by the knockdown of TRF2, which helped us to confirm that autophagy was occurring. Our study also found that knockdown of TRF2 could induce significant telomere dysfunction. Therefore, we speculated that TRF2 deficiency-induced telomere dysfunction might trigger autophagic death, which depends on the activation of the cGAS-STING pathway. However, a more detailed connection between autophagic death and TRF2 still needs further investigation.

Ferroptosis is a novel form of cell death discovered in recent years. An increasing number of studies have shown that promoting ferroptosis is a new way to inhibit the growth of cancer cells [44]. Activation of p53 signalling pathway can promote ferroptosis by repressing the expression of SLC7A11 [45], and telomere dysfunction inevitably activates the p53 signalling pathway [46]. However, it is unclear whether telomere damage in cancer cells promotes ferroptosis through the p53 signalling pathway. Therefore, we used gastric cancer cells as a model to investigate this; we inhibited the expression of TRF2 and then measured the mRNA and protein expression levels of the ferroptosis biomarkers. Our results showed that knockdown of TRF2 in gastric cancer cells resulted in the downregulation of SLC7A11 and GPX4 expressions at the mRNA and protein levels, while the expression of SLC7A11 and GPX4 recovered after pretreatment with the ferroptosis inhibitor ferrostatin-1. Therefore, we believe that inhibition of TRF2 in gastric cancer cells can induce ferroptosis. Additionally, pretreatment with the ferroptosis inhibitor Fer-1 significantly reversed the inhibition of cell growth, proliferation, and migration caused by the knockdown of TRF2, which helped us confirm that ferroptosis was occurring. Next, we wanted to determine whether p53 signalling pathway-mediated ferroptosis was caused by the inhibition of TRF2 in gastric cancer cells. To further study the interaction relationship between TRF2, p53, and SLC7A11, the STRING database was used to analyze their protein interaction network. We found twelve proteins (MDM2, RPA1, UBE3A, EP300, TERF2, BARD1, TP53, GPX4, CREBBP, CDK2, DDX5, and SIRT1) involved in the TRF2, p53, and SLC7A11 protein networks. Based on these results, we speculated that p53-dependent telomere dysfunction might be the bridge mediating the regulation of ferroptosis by TRF2 depletion. The specific mechanism between them needs further investigation in future research.

## 5. Conclusions

In conclusion, we found that TRF2 depletion in gastric cancer cells can inhibit cell growth, proliferation, and migration through the combined action of ferroptosis, autophagic death, and apoptosis, and inhibition of ferroptosis or autophagic death would augment the recovery of cell growth, proliferation, and migration. Our study provides new ideas for the role of telomere protective protein TRF2 in gastric cancer cells, deepens our understanding of the molecular mechanisms underlying the occurrence and development of gastric cancer, and provides new strategies for the treatment of gastric cancer.

## Figures and Tables

**Table 1:** List of proteins in TRF2-p53-SLC7A11-GPX4 interaction network.

Abbreviation of gene	Full name of gene^∗^
AURKA	Aurora kinase A
BARD1	BRCA1-associated RING domain protein 1
CDK2	Cyclin-dependent kinase 2
CREBBP	CREB-binding protein
DDX5	DEAD-box helicase 5
EP300	E1A-binding protein P300
GPX4	Glutathione peroxidase 4
MDM2	MDM2 protooncogene
RPA1	Replication protein A1
SIRT1	NAD-dependent protein deacetylase sirtuin-1
SLC7A11	Solute carrier family 7 (Xc-system), member 11
TERF2	Telomeric repeat-binding factor 2
TP53	Tumour protein P53
UBE3A	Ubiquitin-protein ligase E3A

^∗^The full names of genes are from the GeneCards database (http://www.genecards.org).

## Data Availability

The data used to support the findings of this study are available from the corresponding authors upon request.
